# Brain interleukins and Alzheimer’s disease

**DOI:** 10.1007/s11011-025-01538-5

**Published:** 2025-02-01

**Authors:** Heba G. Abdelhamed, Arwa A. Hassan, Alaa A. Sakraan, Radwa T. Al-Deeb, Dalia M. Mousa, Heba S. Aboul Ezz, Neveen A. Noor, Yasser A. Khadrawy, Nasr M. Radwan

**Affiliations:** 1 Department of Zoology and Chemistry, Faculty of Science, Cairo University, Giza, Egypt; 2 Faculty of Pharmacy & Pharmaceutical Industries, Sinai University, Sinai, Egypt; 3 Department of Zoology, Faculty of Science, Cairo University, Giza, Egypt; 4 National Cancer Institute, Cairo, Egypt; 5 Department of Biotechnology, Faculty of Science, Cairo University, Giza, Egypt; 6 Medical Physiology Department, Medical Research and Clinical Studies Institute, National Research Center, Giza, Egypt

**Keywords:** Interleukins, Alzheimer’s disease, Inflammation, Oxidative stress, Excitotoxicity

## Abstract

The central nervous system (CNS) is immune-privileged by several immuno-modulators as interleukins (ILs). ILs are cytokines secreted by immune cells for cell-cell signaling communications and affect the functions of the CNS. ILs were reported to orchestrate different molecular and cellular mechanisms of both physiological and pathological events, through overproduction or over-expression of their receptors. They interact with numerous receptors mediating pro-inflammatory and/or anti-inflammatory actions. Interleukins have been implicated to participate in neurodegenerative diseases. They play a critical role in Alzheimer’s disease (AD) pathology which is characterized by the over-production of pro-inflammatory ILs. These may aggravate neurodegeneration, in addition to their contribution to detrimental mechanisms as oxidative stress, and excitotoxicity. However, recent research on the relation between ILs and AD revealed major discrepancies. Most of the major ILs were shown to play both pro- and anti-inflammatory roles in different experimental settings and models. The interactions between different ILs through shared pathways also add to the difficulty of drawing solid conclusions. In addition, targeting the different ILs has not yielded consistent results. The repeated failures of therapeutic drugs in treating AD necessitate the search for novel agents targeting multiple mechanisms of the disease pathology. In this context, the understanding of interleukins and their roles throughout the disease progression and interaction with other systems in the brain may provide promising therapeutic targets for the prevention or treatment of AD.

## Introduction

The relationship between the nervous and immune systems has been established with each system modulating the functions of the other system. The neuroimmunological theory focuses on the involvement of the nervous and immune system in several neurodegenerative diseases, including Alzheimer’s disease, Parkinson’s disease and Huntington’s chorea (Reale et al. 2009). Alzheimer’s disease (AD) is the most prevalent of the known neurodegenerative disorders and the underlying cause of dementia in old adults worldwide (Alzheimer’s Association 2023). It is characterized by progressive deterioration in memory. Its major neuropathological hallmarks are the deposition of extracellular β-amyloid (Aβ) plaques produced from amyloid precursor protein (APP) by proteolytic cleavage and the intracellular Tau protein neurofibrillary tangles formed by phosphorylation (Long and Holtzman 2019).

However, neuronal damage and neuropathologic lesions occur in several brain regions before AD is diagnosed (DeKosky and Marek 2003) making it challenging to investigate the early onset stages and the pathogenic pathways involved in disease development and progression. The development of AD passes through three stages: the pre-symptomatic stage, the prodromal stage characterized by mild cognitive impairment (MCI), and the clinical form of AD (Reitz et al. 2011).

The first clinical manifestations of AD are mild memory deficits which progress to complete cognitive impairment with disturbances in language, behavior and judgment (Pless et al. 2023). Aβ accumulation is complex and associated with failure of microglial activity to remove it (Plantone et al. 2024). In addition, other pathways participate in AD pathogenesis, including neuronal damage, neuroinflammation, excitotoxicity, and oxidative stress (von Bernhardi and Eugenín 2012; Morales et al. 2014). However, the pathological sequences and the interaction of these different mechanisms at the early stages and throughout the course of the disease are not clear. Neuroinflammation has both protective and damaging effects. At the onset of the disease, inflammation is primarily mediated by activated microglia and astrocytes within and around senile plaques (Ozben and Ozben 2019) which induces the release of numerous pro-inflammatory cytokines and other inflammatory products together with reactive oxygen species (ROS) (Morales et al. 2014; Rani et al. 2023). Although activation of microglia at early stages facilitates phagocytosis of Ab plaques and maintains neuronal survival, chronic inflammation becomes skewed toward a proinflammatory pattern, which might be neurotoxic (Del-Aguila et al. 2019). Prolonged chronic inflammation sustains elevated levels of cytokines and chemokines severely impacting neuronal survival (Li et al. 2023). Furthermore, Aβ itself can trigger the expression of several pro-inflammatory cytokines, such as TNF-α, IL-6, interferon-γ (IFN-γ) and IL-1β by glial cells, and several chemokines, including MCP-1, IL-8, CXCL10 (IP-10), and CCL5, increasing the recruitment of immune cells from the peripheral blood (Domingues et al. 2017). Immune cells can cross the BBB exaggerating neuroinflammation in the brain (Fiala et al. 1998). Sustained inflammation may also facilitate tau phosphorylation, exacerbating the neuronal damage (Lee et al. 2010).

The recent theory of homeostasis network collapse suggests that the imbalance between excitation and inhibition in the brain results in dysregulation of neuronal networks. This may be a causative factor or even exacerbate the pathogenesis of AD (Frere and Slutsky 2018; Vico Varela et al. 2019). Novel research and experimental approaches showed that the microglial population was heterogeneous and emphasized the complexity of their function and dysfunction (Jesudasan et al. 2021; Leng and Edison 2021). Microglia express several neurotransmitter receptors for glutamate, GABA, acetylcholine, dopamine, adrenalin, ATP, and adenosine which suggests that neurotransmitters play a crucial role in regulating microglial function (Stolero and Frenkel 2020). At the same time, microglia may influence glutamatergic neurotransmission (Ben Achour and Pascual 2010).

It has been reported that the proinflammatory cytokines released at the neurodegenerative site in the brain stimulated damage-associated molecular patterns (DAMPs) and/or pathogen-associated molecular patterns (PAMPs) and other receptors generally found in AD brains (Roh and Sohn 2018) leading to apoptosis. Translocator protein (TSPO) PET studies in both humans and mice showed two peaks in the AD pathogenesis: an earlier peak associated with neuroprotection, and a later peak that appears when the disease worsens, and is correlated with neurotoxicity (Hamelin et al. 2016; Focke et al. 2019). This suggests that novel therapies should not only concentrate on inflammation suppression but must also shift microglia from the neurotoxic to the neuroprotective phenotype (Cummings et al. 2020).

The present review provides an overview of the different interleukins and their conflicting roles as pro- and/or anti-inflammatory agents in AD neuroinflammation. It emphasizes the correlation between interleukins, oxidative stress and excitotoxicity in the pathogenesis of AD. The development of new therapeutic agents that target these interleukins in AD has also been summarized. However, most of the existing therapies focus on one interleukin and one aspect only of the underlying pathology and ignore the interaction between the different interleukins on one hand and between interleukins and other related pathologies on the other hand. The aim of this review is to throw light on the importance of investigating all the neurochemical-immunological alterations in AD pathogenesis to take them into consideration when developing novel potential therapies against AD.

## Interleukins and Alzheimer’s disease

Despite the controversy about the role played by neuroinflammation in AD initiation, it is now widely accepted that the immune system participates in the pathogenesis of AD (Taipa et al. 2018). Microglia are normally present in an inactive state. If microglia are activated by lipopolysaccharide (LPS), IFN-γ or TNF-α, they are considered ‘‘classically activated’’ (M1) (Boche et al. 2013). M1 microglia are responsible for the defense against pathogens and tumor cells by releasing proinflammatory cytokines and free radicals and are thus associated with neuronal damage. Conversely, the M2 form is the alternative anti-inflammatory form concerned with enhancement of tissue remodeling and/or repair and stimulation of angiogenesis by producing anti-inflammatory cytokines (Czeh et al. 2011). Mawuenyega et al. (2010) proposed that the inability of the immune system to eliminate Aβ, not the accumulation of this peptide, is the mechanism underlying the etiology of sporadic AD. The microglia and mononuclear phagocytes fail to confine Aβ aggregation and transform into a chronic pathologic activated form (Heneka et al. 2010), with cytokines overproduction (Sochocka et al. 2017) in the brain and CSF (Blennow et al. 2010).

**Fig. 1:**
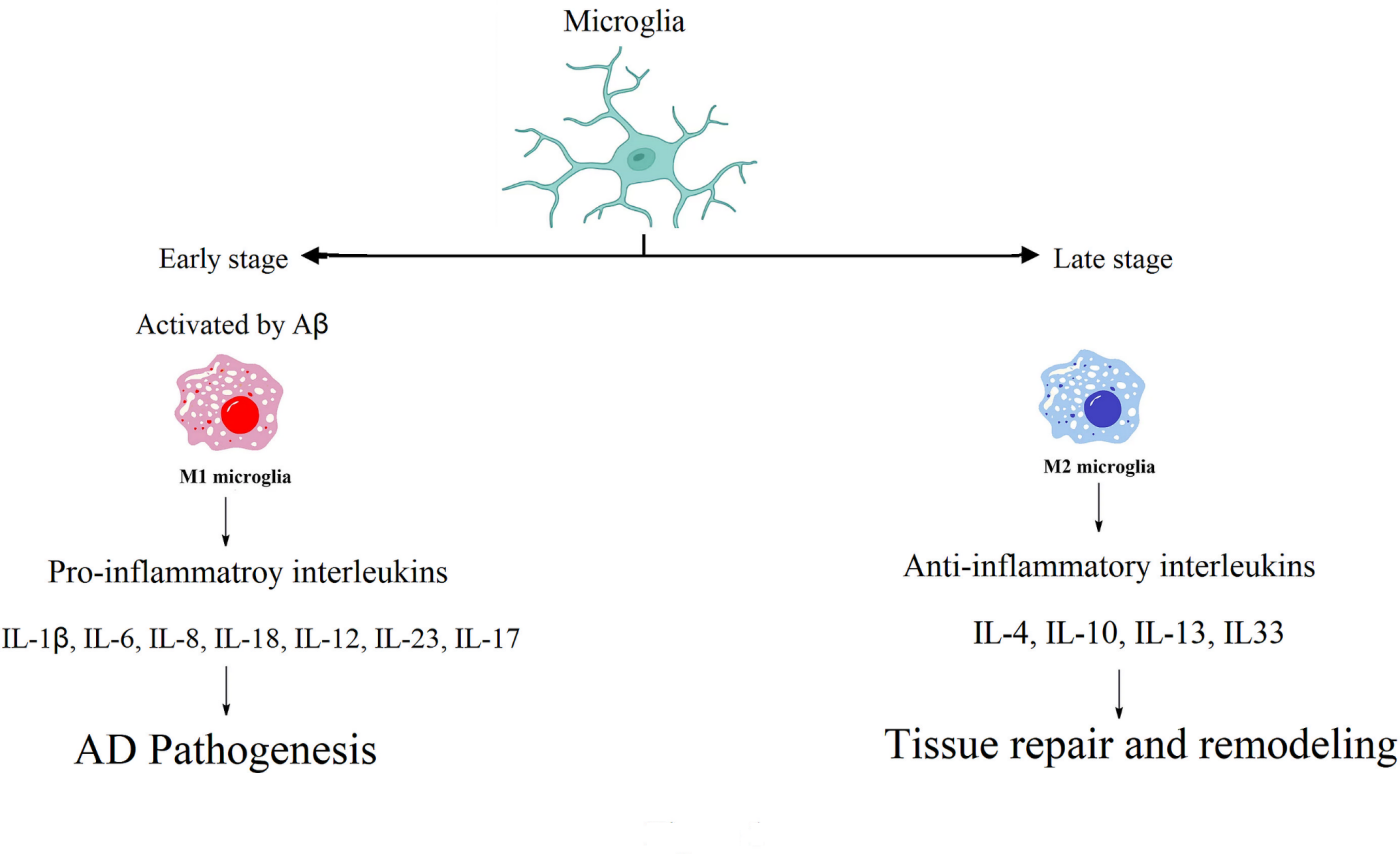
Microglial phenotypes and functions in AD stages

In AD mouse models, Wang et al. (2015) reported an increase in both M1 (TNF-α,IL-1β, IL-6 and iNOS) and M2 (IL-10, YM1, Arg-1, and Mrc) markers. A progressive switch occurs changing microglia from a neuroprotective to a neurotoxic phenotype throughout the course of AD pathology. Aβ binds to the cell surface of microglia increasing the gene expression of pro-inflammatory cytokines as TNF-α, IL-1β, IL-6, IL-18; which in turn enhance tau hyperphosphorylation causing neuronal death (Von Bernhardi et al. 2010) and initiating a vicious circle (Fig. 1).

### Current evidence from clinical studies

**Fig. 2:**
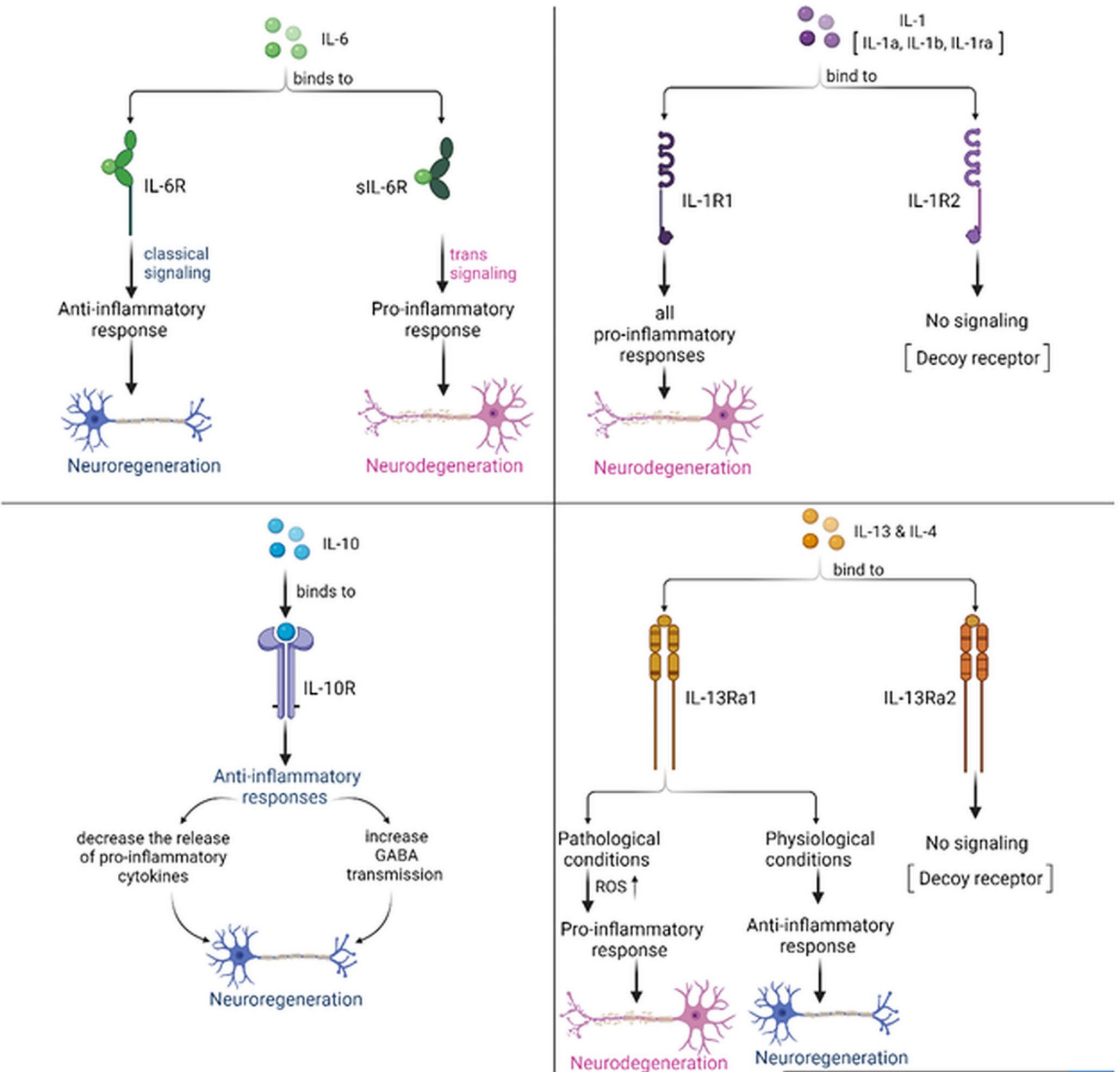
The actions of the pro-inflammatory ILs, IL-1 and IL-6, and the anti-inflammatory ILs, IL-10, IL-4 and IL-13

Microglial activation has been reported in AD patients and animal models (Cagnin et al. 2001). It is associated with increased release of pro-inflammatory cytokines as TNF-α, IL-1β, IL-6, IFN-γ, IL-18, and upregulation of brain cytokine receptors as evident from experimental and clinical studies (Sastre et al. 2006; Ojala et al. 2009). The upregulation of the proinflammatory cytokine IL-1β is considered an early indicator of AD (Lopez-Rodriguez et al. 2021). IL-1β release from microglia is stimulated by Aβ during AD onset (Facci et al. 2018). During AD progression, the activated microglia cluster around Aβ plaques to enhance their phagocytosis. Increased plasma levels of IL-1β, IL-1Ra, IL-33, IL-18BP, and soluble receptors sIL-1R1, sIL-1R3, and sIL-1R4 have been recorded in patients with AD (Italiani et al. 2018) suggesting that systemic chronic inflammation is associated with AD pathogenesis. In the early AD stages, activated microglia upregulate IL-12 and IL-23 secretions which transform the microglia to a pathogenic state with impaired Aβ clearance (Nitsch et al. 2021). IL-12 members also modulate other immune cells to secrete pro-inflammatory interleukins which further increase the severity of AD. IL-23 induces Th17 cell differentiation to form IL-17 which plays a critical role in AD onset and progression triggering the onset of synaptic and cognitive deficits in the early stages of the disease (Brigas et al. 2021). Genetically, IL-6 gene overexpression has been observed in patients with late AD (Griciuc et al. 2019) suggesting that IL-6 affects AD progression and severity. It is clear that the interactions between specific interleukins can participate in the activation of specific immune cell populations which in turn release other interleukins all of which may enhance AD pathogenesis through shared pathways. On the other hand, anti-inflammatory cytokines, such as IL-1ra, IL-33, and IL-10, are significantly increased in the CSF and plasma in AD, reducing neuroinflammation (Brosseron et al. 2014) (Fig. 2).

## Role of interleukins in neuroinflammation

### Interleukin-1 and Alzheimer’s disease

The upregulation of IL-1 has been implicated to participate in neurodegeneration early in AD development and in AD mice models. IL-1 is critical for β-amyloid plaque deposition and its expression was found to increase in AD (Bona et al. 2008). Several studies reported an over-expression of IL-1β which is released by microglia and astrocytes and accumulates around Aβ plaques in the brain and animal models of AD (Hunter et al. 2012; Boutajangout and Wisniewski 2013). An elevation in IL-1β due to specific polymorphisms was found in AD patients suggesting that IL-1β production begins early and is enhanced throughout the progression of the disease (Forlenza et al. 2009). Activated microglia and astrocytes produce IL-1β in an inactive pro-form, termed pro-IL-1β, which is converted to the active form after cleavage by caspase-1 (Lamkanfi and Dixit 2012; Liu et al. 2013). Parajuli et al. (2013) reported that soluble amyloid β enhances the transformation of pro-IL-1β to active IL-1β in the microglial cells. Increased serum IL-1β levels are used as a marker for neurodegeneration to distinguish between normal ageing and AD (Forlenza et al. 2009).

Moreover, IL-1β has been shown to enhance amyloid precursor protein (APP) expression in neuronal cell culture. The cleavage of APP by the enzymes β- and γ-secretase yields Aβ. In addition, IL-1β overexpression exaggerates tau phosphorylation and formation of neurofibrillary tangle by activating p38-MAPK and glycogen synthase kinase 3 (GSK3) (Kitazawa et al. 2011). These affect synaptic plasticity and impair learning and memory (Pickering and O’Connor 2007). Free Aβ activates microglia, causing overproduction of pro-inflammatory cytokines as IL-1β and reactive oxygen species (ROS) (Rubio-Perez and Morillas-Ruiz 2012). Accordingly, it has been proposed that Aβ aggregation can be a cause and/or a consequence of enhanced IL-1β expression in patients with AD. In addition, increased IL-1β expression was found to impair the ability of microglia to clear Aβ (Heneka et al. 2010) and increase BBB permeability, thus enhancing Aβ accumulation in the brain (Wang et al. 2014).

Contrary to the above findings, some authors presented evidence that IL-1β may play a role in reducing AD pathology. Several authors reported that sustained IL-1β overexpression decreases Aβ-induced pathology by regulating the degradation of microglial-dependent plaque or enhancing the cleavage of non-amyloidogenic APP in a mouse AD and a cell culture model (Shaftel et al. 2007; Tachida et al. 2008; Ghosh et al. 2013). Thus, IL-1β may have a complex role in AD pathogenesis. However, it is mostly accepted that the proinflammatory IL-1β effects enhance disease impairment through numerous factors and pathways (Xie et al. 2015). Supporting the role of IL-1 in AD, IL-1 signaling inhibition in transgenic AD mice by IL-1R knockout was found to reduce the burden of Aβ (Williamson et al. 2011) and this protective impact was believed to depend on decreased AD-related neuroinflammation (Barrientos et al. 2012).

On the other hand, IL-1α upregulation has been demonstrated as an early event in mild cognitive impairment (MCI) CSF. Evidence suggests that IL-1α is important in AD staging and may be considered a novel biomarker for the early detection of AD pathology (Hu et al. 2010).

### Interleukin-2 and Alzheimer’s disease

It has been reported that serum IL-2 levels decreased in patients with AD, in comparison with elderly subjects (Beloosesky et al. 2002). It was found that IL-2 knockout mice suffered from impaired learning and memory which coincided with cytoarchitectural alterations in the hippocampal dentate gyrus (Beck et al. 2005). Alves et al. (2017) reported that elevated IL-2 in the hippocampus of APP/PS1ΔE9 mice treated with IL-2 increased Treg activation and improved AD pathology suggesting that the retrieval of memory impairment in these animals was mediated by IL-2-induced tissue remodeling, increased synaptic plasticity and recovery of spine density. In addition, IL-2 treatment and subsequent astrocytic activation decreased amyloid plaques and reduced the ratio of amyloid-b42 to amyloid-b40 (Murray et al. 2012). The elevated amyloid-b40 in APP/PS1ΔE9 mice treated with IL-2 protected neurons from amyloid b42-induced brain damage (Zou et al. 2003) and inhibited amyloid deposition in AD mice (Na and Kim 2007). In another mouse model of AD (APP/PS1 mice) treated at early stages with low-dose IL-2, IL-2 was proposed to enhance cortical plaque-associated microglial activation (Dansokho et al. 2016).

### Interleukin-3 and Alzheimer’s disease

IL-3 is a multifunctional cytokine that contributes to inflammatory disorders and autoimmune diseases (Mindur and Swirski 2019). IL-3 levels were correlated with risk (Soares et al. 2012) and severity (Kiddle et al. 2012) of AD in humans. In vitro studies implicated IL-3 in neurodegeneration (Zambrano et al. 2007, 2010). Recently, McAlpine et al. (2021) suggested that IL-3 mediates astrocyte–microglia communication that regulates microglial programming, and protects against AD. The authors found that the injection of rIL-3 into the cortex of 5xFAD mice induced a rapid microglial mobilization with microglial accumulation around Aβ. They concluded that IL-3 has a therapeutic efficacy in AD and could be used as a potential target to reduce AD pathology.

### Interleukin-4, Interleukin-13 and Alzheimer’s disease

**Fig. 3:**
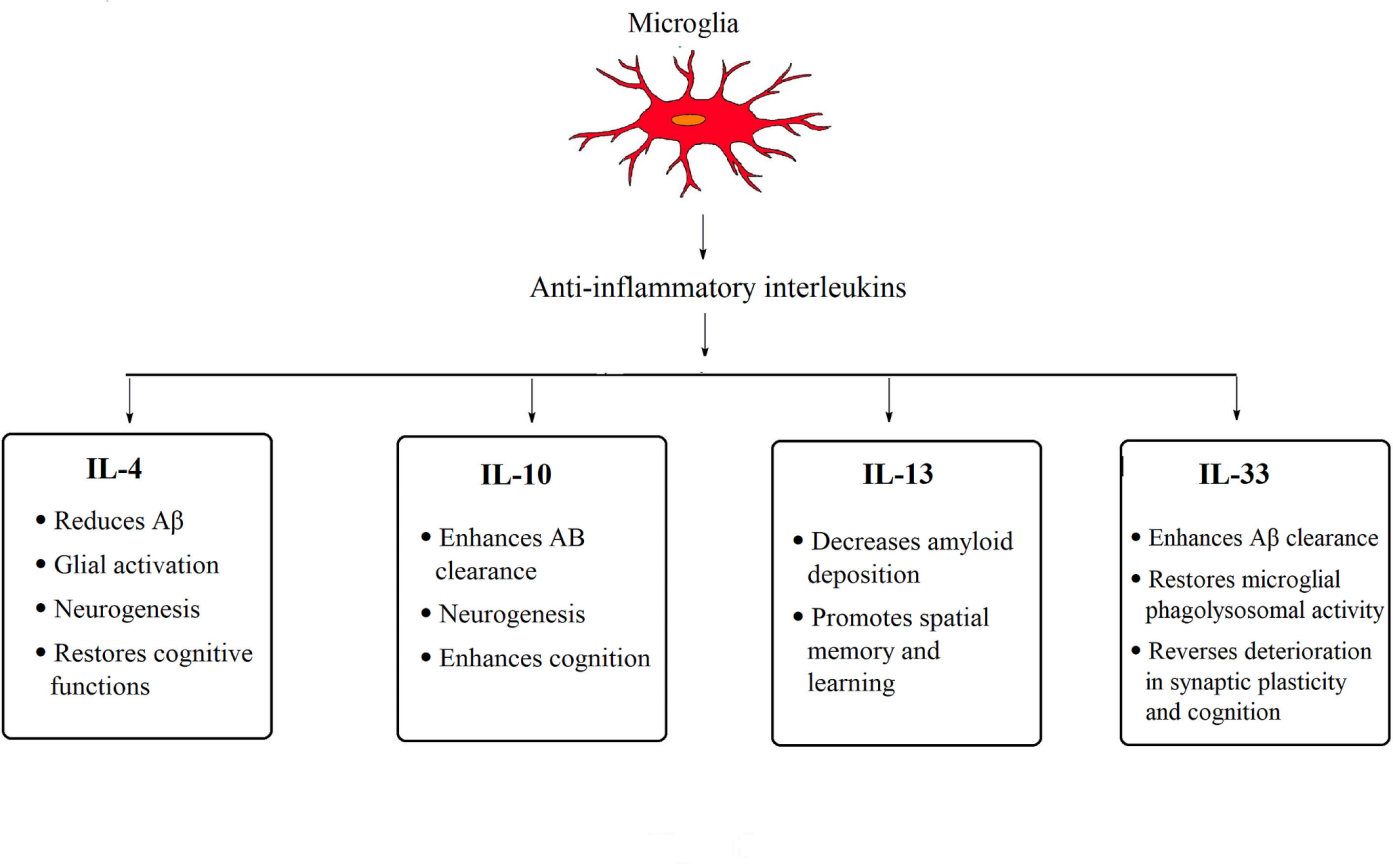
The role of anti-inflammatory ILs in AD

There is accumulating evidence that IL-4 and IL-13 have neuroprotective effects in transgenic AD mouse models. Intracerebral injection of both IL-4 and IL-13 decreased amyloid deposition and promoted spatial memory and learning in transgenic mice (Kawahara et al. 2012). An obvious correlation between single IL-4 gene nucleotide polymorphisms and AD risk was evident (Ribizzi et al. 2010) with a reduction in IL-4 formation in peripheral mononuclear cells in the blood of AD patients (Reale et al. 2008). Moreover, Gonzalez-Dominguez et al. (2015) reported that reduction of IL-4 may promote the pathology of AD in the APP/PS1 mouse model. Several studies proposed that the increase in IL-4 expression mediates the action of acetylcholinesterase inhibitors in AD patients (Gambi et al. 2004; Reale et al. 2004), suggesting that this interleukin has an immunomodulatory role in AD (Fig. 3).

Treatment of rat hippocampus with IL-4 reduced long-term potentiation (LTP) impairment and IL-1β expression caused by Aβ in rat hippocampus (Lyons et al. 2007). Moreover, stimulation of human macrophages or microglia by IL-4 increased Aβ degradation (Shimizu et al. 2008). Several studies reported a beneficial role for IL-4 on brain homeostatic functions with amelioration of AD symptoms by reducing inflammation and providing a favorable environment (Maher et al. 2005; Lyons et al. 2007; Gadani et al. 2012). Kiyota et al. (2010) demonstrated that neuronal IL-4 expression reduced Aβ, glial activation, and neurogenesis thus restoring cognitive functions in APP + PS1 mice. A single IL-4 intrahippocampal injection reduced Aβ plaque density in APP/PS1 mice after 5 days (Cherry et al. 2015). Tang et al. (2019) reported that microglia pretreated with IL-4 induced autophagic flux, inhibited the blockade of Aβ-induced autophagic flux and enhanced Aβ uptake and degradation. The authors suggested that this may be considered a novel mechanism through which IL-4 performs its protective effects in AD.

IL-13, another ligand of the IL-4 receptor, showed similar results (Kawahara et al. 2012). Barroeta-Espar et al. (2019) found increased IL-13 and IL-4 levels with reduced gliosis and neuronal damage in the entorhinal cortex of patients exhibiting AD pathology. Furthermore, injection of a mixture of IL-4 and IL-13 decreased brain Aβ and enhanced cognitive ability in APP23 transgenic mice (Kawahara et al. 2012).

By contrast, IL-4 and/or IL-13 were reported to potentiate Aβ-induced inflammation by generating ROS and proinflammatory cytokines as TNF-α, IL-6 and inducible nitric oxide synthase (iNOS) (Nam et al. 2012; Park et al. 2009).

### Interleukin-6 and Alzheimer’s disease

The molecular mechanisms underlying the relation between IL-6 and AD have been extensively investigated. IL-6 manipulation revealed both neurodegenerative and neuroprotective effects according to neuronal condition. Initial stimulation of IL-6 displayed a destructive effect, however later, the extensive neurodegeneration induced a neuroprotective effect of IL-6. It has been shown that IL-6 levels were increased mostly in the early stages of AD due to plaque formation (Wang et al. 2015). Activation of microglia and other glial cells by Aβ depositions induced the release of IL-6 (Sondag et al. 2009). IL-6 resulted in memory impairment, as inhibition of IL-6 enhanced long-term potentiation and improved long-term memory in a hippocampal-dependent task (Balschun et al. 2004). It has been shown that IL-6 increased in the plasma, CSF and brain of AD patients (Brosseron et al. 2014; Lyra e Silva et al. 2021). IL-6 was found to participate in early-stage amyloid plaque formation in AD brains (Huell et al. 1995) by stimulating amyloid precursor protein (APP) synthesis (Ringheim et al. 1998) and tau phosphorylation (Quintanilla et al. 2004). It also underlied the synaptic loss and learning deficits in mice (Quintanilla et al. 2004; Burton and Johnson 2012). It has been clearly established that IL-6 contributed to the processing and production of APP in rat primary cortical neurons (Spooren et al. 2011). Free Aβ, in turn, activates microglia, increasing the synthesis and secretion of IL-6 (Vukic et al. 2009). Furthermore, intracerebroventricular (ICV) Aβ injection elevated peripheral IL-6 (Song et al. 2001), whose trans-signaling could increase APP transcription (Ringheim et al. 1998). Moreover, the neuronal damage caused by Aβ in cultured rat cortical neurons was enhanced by IL-6 (Qiu and Gruol 2003). IL-6-treated hippocampal cells participated in NFT formation by Tau hyperphosphorylation through pathway deregulation, cd k5/p 35 or other pathways as JAK/STATs, NMDA receptors and the MAPK-p38 protein kinases (Spooren et al. 2011).

However, there is some controversy in the literature. Although evidence confirm a synergistic relationship between Aβ neurotoxicity and IL-6 levels, in vivo studies reported that IL-6 overexpression suppressed Aβ deposition in APP transgenic mice (Chakrabarty et al. 2010). The authors suggested that attenuation of Aβ levels in IL-6-induced neuroinflammation may be due to microglial activation to a beneficial (M2) phenotype which increased the phagocytosis and clearance of Aβ.

### Interleukin-8 and Alzheimer’s disease

It has been demonstrated that, in cultured human microglia, IL-8 may have a crucial role in inducing pro-inflammatory reactivity in AD. A comprehensive microarray analysis examined the expression of several pro-inflammatory chemokines and cytokines after exposure of cortical microglial to a low dose of amyloid-beta (Aβ) and revealed that IL-8 expression showed the largest up-regulation of the genes analyzed (Walker et al. 2001).

It has been reported that Aβ peptide increased IL-8 levels in a dose-dependent manner in diseased and control human microglia. CSF IL-8 levels were elevated in mild cognitive impairment (MCI) and AD-patients (Galimberti et al. 2006). The authors suggested that increased IL-8 in MCI patients was clinically important since MCI may be a risk for the development of Sokolova et al. (2009) found that IL-8 and monocyte chemo-attractant protein 1 (MCP-1) levels were elevated in the brain of AD patients. Alsadany et al. (2013) reported that the increased IL-8 levels in AD patients were associated with a reduced performance in cell cognition tasks.

### Interleukin 9 and Alzheimer’s disease

Although changes in IL-9 have not been consistently reported in AD (Stertz et al. 2018), evidence showed that knockin APOE ε4 allele in mice induced higher IL-9 formation than the wild-type ε3 allele (Mace et al. 2007). Moreover, Upadhya (2020) reported that IL-9R expression was up-regulated in AD.

### Interleukin-10 and Alzheimer’s disease

A*β* was shown to be inhibited in glial cells pre-exposed to IL-10 (Szczepanik et al. 2001), which suggested the presence of IL-10 receptors on cultured glial cells (Ledeboer et al. 2003). IL-10 inhibited the release of TNF-*α*, GMSF, MIP-1*α*, MIP-2*α*, IL-1, IL-6, IL-8 and IL-12 by monocyte and macrophages (Clarke et al. 1998). The reduced expression of anti-inflammatory cytokines, as IL-10, enhanced the susceptibility of patients to develop AD (Su et al. 2016). In this context, the overexpression of hippocampal IL-10 in AD transgenic mice has been shown to enhance neurogenesis and cognition, thus providing evidence that IL-10 provides neuroprotection against AD pathology (Kiyota et al. 2012). Recently, Weston et al. (2021) reported that IL-10 deficiency can promote tau hyperphosphorylation, enhance pro-inflammatory cytokine expression, and activate microglial morphology in AD-relevant tau epitopes.

In contrast, Mun et al. (2016) demonstrated that removal of IL-10 reduced synaptic and cognitive impairments and enhanced the clearance of Aβ in knockout mice. In addition, peripheral interleukin IL-10 was associated with brain atrophy and was proposed as a potential biomarker for evaluating the extent of disease progression (Magalhães et al. 2017; Park et al. 2020).

### Interleukin 15 and Alzheimer’s disease

It has been reported that serum IL-15 levels were negatively correlated with neuropsychiatric symptoms in AD patients (Hall et al. 2013). It was found that IL-15 was correlated with basic daily life activities in AD patients in a gender-dependent manner (Hall et al. 2012). In addition, AD patients had elevated CSF IL-15 levels (Rentzos et al. 2006) which decreased after treatment with acetylcholinesterase inhibitors (Rentzos et al. 2007). Blocking IL-15 in cultured microglia decreased the activation of inflammatory mediators as nuclear factor (NF)-κB and mitogen-activated protein kinases (MAPK) (Gómez-Nicola et al. 2008). Bishnoi et al. (2015) suggested that serum IL-15 was correlated with dementia in AD.

### Interleukin-12 and IL-23 and Alzheimer’s disease

A major inflammatory signaling pathway in AD pathology involves IL-12 and IL-23. IL-12 levels were elevated in brain and CSF (Guerreiro et al. 2007) while IL12p40 levels were de-regulated in the CSF of AD patients (Vom Berg et al. 2012), suggesting a relation between plasma IL12p40 and AD (Hu et al. 2012). Increased IL12p70 levels were also found in brain of AD patients, emphasizing the importance of IL-12 in AD (Wood et al. 2015). Recently, Eede et al. (2020) reported that IL-12 and IL-23 were up-regulated by microglia in the brain of AD-like APPPS1 mice. In transgenic mouse models of amyloidosis, when the shared p40 subunit is removed genetically or blocked pharmacologically with specific antibodies, both IL-12 and IL-23 signaling were inhibited causing a decrease in AD pathology (Vom Berg et al. 2012; Eede et al. 2020).

In a northern Han Chinese population, single nucleotide polymorphisms were induced in the IL-12/23 subunit p40 (rs3212227) (Zhu et al. 2014) and IL-23 receptor polymorphisms were correlated with AD (Liu et al. 2014). AD patients showed higher peripheral IL-23 levels (Chen et al. 2014a, b) and the p40 subunit concentration was considered a serum biomarker through which the Aβ load could be predicted in AD (Pedrini et al. 2017). The functional importance of IL-23 in AD has been suggested from mice models as the APP/PS1model in which Aβ plaques developed after 6–8 months, with no other aspects of AD pathology (Myers and McGonigle 2019). In addition, preclinical findings revealed the significant role played by IL-23 in neuroinflammation and formation of plaques in AD models and proposed anti-IL-23 treatment as a potential treatment approach in AD (Nitsch et al. 2021). Recently, it has been reported that IL-12, but not IL-23, underlied AD-specific IL-12/IL-23 neuroinflammation, which preferentially targeted oligodendrocytes and neurons expressing IL-12 receptors (Schneeberger et al. 2021).

### Interleukin 17 and Alzheimer’s disease

IL-17 has been suggested to participate in AD pathology (Zenaro et al. 2015). The circulating Th17 cells were increased in patients suffering MCI (Oberstein et al. 2018), with an increase in serum IL-17 levels during AD progression (Chen et al. 2014a). It has been found that Th17 cells infiltrated the brain of AD animal models, resulting in neuroinflammation and death of neurons (Siffrin et al. 2010; Yang et al. 2017). Over-expression of IL-17 may result from a destructive mutation of TLR4 and elevated IL-1b levels in AD mice brain homozygous (Jin and Dong 2013). IL-17 neutralization was reported to alleviate inflammation and memory disorders caused by Aβ treatment (Cristiano et al. 2019).

However, the involvement of IL-17 in early AD stages is controversial. Brigas et al. (2021) described a pathogenic role of IL-17 in enhancing synaptic and cognitive dysfunction during the onset of AD and provided evidence that IL-17 can have a dual role that depends on its concentration, with the high levels leading to severe inflammation. The authors found that the cells releasing IL-17 accumulated in the meninges and brain at the early AD stages and suggested that the pathophysiological dysregulation of IL-17 levels promoted neurodegeneration. Th17 cells form IL-17. IL-22 can disrupt the BBB leading to the infiltration of Th17 cells. Th17 cells may injure neurons directly by the Fas/FasL pathway in the AD rat model induced by Aβ (Zhang et al. 2013a, b).

However, the interaction between IL-17 and IL-23 is not clear as both interleukins contribute to AD-associated neuroinflammation (Vom Berg et al. 2012). It has been proposed that IL-23 might enhance the development and expansion of Th17 and increase IL-17 formation (Aggarwal et al. 2003). Chen et al. (2014a) observed increased IL-18, IL-23 and IL-17 levels in Chinese patients suffering from AD.

Neuroinflammation mediated by Th17 cell contributed to the neurodegeneration induced in a rat model of AD (Zhang et al. 2013a, b). Furthermore, Saresella et al. (2014) demonstrated that Th17 cell activation occurred in AD patients and suggested that infiltration of Th17 cells into the brain played a role in the neuroinflammation and neurodegeneration of AD by proinflammatory cytokine release and by a direct action through the Fas/FasL apoptotic pathway (Zhang et al. 2013a, b). Moreover, Mohammadi Shahrokhi et al. (2018) reported that IL-23 and IL-17 A, acted as biomarkers of Th17 cells, and confirmed the neuroinflammatory inducer role of IL-17 A in AD. Cao et al. (2023) suggested that IL-17 A may promote the formation of Aβ plaques, enhance the release of TNF-α by microglia and reduce the phagocytic activity of microglia thus affecting AD pathogenesis. The authors reported that IL-17 A inhibition reduced Aβ deposition in the brains of AD patients, reducing neuroinflammation, and delaying AD progression.

Conversely, IL-17 A overexpression was proposed to be neuroprotective in AD mouse brain as it reduced amyloid angiopathy and relieved memory and learning impairments (Yang et al. 2017).

### Interleukin-18 and Alzheimer’s disease

Accumulating evidence suggest the involvement of IL-18 in AD. This was confirmed by the increase in IL-18 levels in AD brains (Ojala et al. 2009), and diseases that increase AD risk, as T2DM (Aso et al. 2003), ischemic heart disease (Mallat et al. 2001), obesity (Esposito et al. 2002) and cognitive impairment (Bossù et al. 2010). Moreover, the intracellular caspase-1 responsible for IL-18 activation was up-regulated in brain tissues from AD patients (Pompl et al. 2003). The enhancement of IL-18 expression in AD brain may be partly caused by its long half-life (Ojala et al. 2009).

IL-18 can increase Aβ production (Sutinen et al. 2012) and the kinases glycogen synthase kinase-3β (GSK-3β) and Cdk5, which contribute to tau hyperphosphorylation (Ojala et al. 2008). Moreover, IL-18 can induce IFN which triggers immunoinflammatory processes, and induces indoleamine 2,3-dioxygenase (IDO). The stimulation of IDO diverts the metabolism of tryptophan to the kynurenine pathway (KP), whose products, as quinolinic acid, can be excitotoxic and/or diabetogenic, and participate in AD pathology (Anderson and Ojala 2010).

Therefore, increased IL-18 levels can play a significant role in AD pathogenesis, either directly or via oxidative stress as it modulates several antioxidant enzymes (Sutinen et al. 2014). In addition, it can moderately increase tau hyperphosphorylation and modulate anti-apoptotic B-cell lymphoma-extra-large (Bcl-xL) protein levels (Sutinen et al. 2012). The authors reported that binding of IL-18 to its receptor complex can activate JNK and MAPK p38, which can stimulate the pathways of both extrinsic and intrinsic pro-apoptotic signaling, suggesting that IL-18 may induce apoptosis leading to the progression of AD. They suggested that up-regulation of IL-18 expression can cause an adverse inflammatory vicious cycle in which IL-18 induces the formation of IL-1β and IFN-γ, the latter cleaving inactive IL-1β and IL-18 to produce their mature active forms by caspase-1, thus enhancing IL-18 expression which participates in the pathogenesis of AD. The latter authors found that, in differentiated SH-SY5Y cells in human, IL-18 increased the production of APP and its Thr668 phosphorylation and also enhanced Aβ by increasing the expression of the N-terminal fragment (NTF) of PS-1 which belongs to the functional γ-secretase complex.

On the other hand, it has been shown that IL-18 was cytotoxic to cardiomyocytes, as it increased intracellular Ca^2+^ levels, and calcium dysregulation which participate in AD pathogenesis (Yu et al. 2009). More importantly, IL-18 inhibited LTP induction in the dentate gyrus, a mechanism underlying learning and memory (Curran and O’Connor 2001). However, low IL-18 levels due to interleukin gene polymorphisms may be associated with better physical functions in healthy aged men, suggesting a neuroprotective effect (Frayling et al. 2007). Bossù et al. (2007) found a relationship between gene promoter polymorphisms in IL-18 and the susceptibility and therapeutic recovery of AD.

IL-18 and reactive oxygen species (ROS) can also regulate the blood brain barrier (BBB) through increasing the expression of several matrix metalloproteinases (MMPs) enhancing BBB permeability. This increased the influx of brain inflammatory cytokines and ROS, and affected AD progression (McColl et al. 2010). These multiple changes might also cause caspase-1 activation and neuronal death. Several AD susceptibility genes may interact with these processes, both centrally and systemically (J. O. Ojala and Sutinen 2017).

### Interleukin-33 and Alzheimer’s disease

IL-33 has been reported to possess both pro- and anti-inflammatory properties, as evident from conflicting data showing both increased and decreased IL-33 in AD. Cellular and genetic animal and human studies supported the use of IL-33 as a therapeutic target in AD (Carlock et al. 2017; Fu et al. 2016). It has been suggested that microglia lose their ability to phagocytoze Aβ during AD progression (Hickman et al. 2008). In an animal study using AD mouse models, peripheral IL-33 administration was shown to decrease the levels of soluble Aβ and the deposition of amyloid plaque and reverse the deterioration in synaptic plasticity and cognitive functions (Fu et al. 2016). The authors found that IL-33 treatment enhanced Aβ uptake by microglia in APP/PS1 mice and suggested that IL-33 could restore the phagolysosomal activity of microglia and enhance Aβ clearance. Moreover, Carlock et al. (2017) reported that IL-33 reduction induced tau abnormality, AD-like symptoms, and neurodegeneration in aged mice. Similarly, genetic study in human found reduced IL-33 expression in AD brains and revealed three single nucleotide polymorphisms in the gene of IL-33 showing a decreased vulnerability for the development of AD (Chapuis et al. 2009). The authors suggested that IL-33 has a potential therapeutic role in AD. Saresella et al. (2020) found reduced circulating IL-33 levels in AD patients and attributed this reduction to the increased decoy receptor sST2 in the blood of AD patients. In IL-33-deficient aged mice, abnormal tau accumulation and late-onset neurodegeneration were observed in the cerebral cortex and hippocampus and were accompanied by impaired memory (Cao et al. 2018).

However, Xiong et al. (2014) reported the presence of high concentrations of IL-33 in the neuropathological lesions of AD brain and suggested that IL-33 can aggravate neuroinflammation in AD.

## Role of interleukins in excitotoxicity

**Fig. 4:**
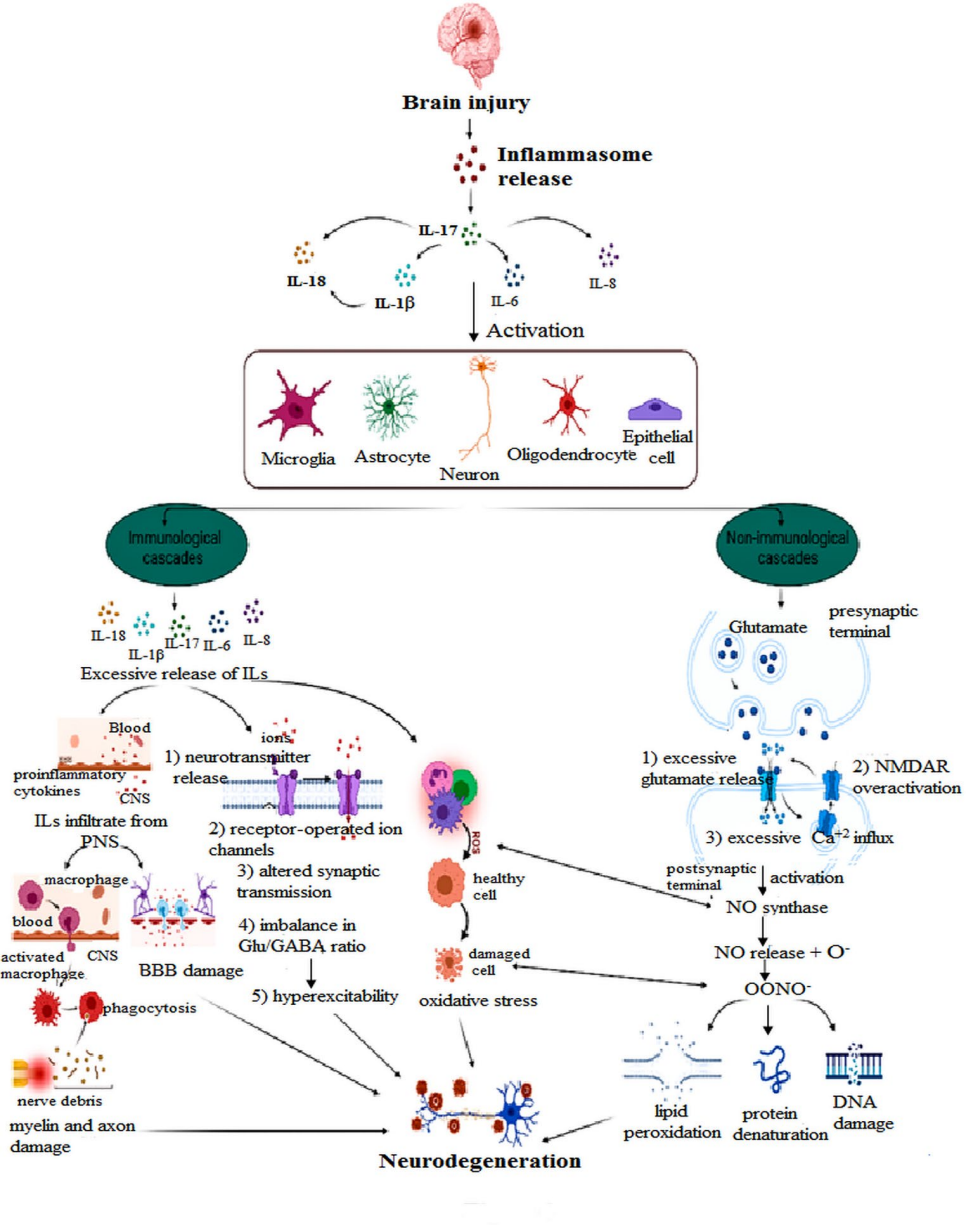
The different mechanisms caused by brain injury to result in neurodegeneration. Any insult in the brain results in inflammation, where IL-17 release causes other ILs to be released like IL-1, IL-6, IL-8 and IL-18 that act on their receptors to activate different neuronal and immune cells to stimulate two different cascades: immunological and non-immunological. The immunological cascade causes inflammation progression which results in 1. Myelin & axon damage, 2.BBB damage, 3. Hyperexcitability and 4. Oxidative stress. The non-immunological cascade affects glutamate release causing peroxynitrite formation that results in 1. Lipid peroxidation, 2. Protein denaturation and 3. DNA damage. Finally, all these events result in neurodegeneration

Glutamate is the major excitatory neurotransmitter in the mammalian CNS. Glutamate concentrations must be maintained within a low physiological range to protect neurons from excitotoxic damage (Dong et al. 2009). It has been established that there is a correlation between interleukins and glutamate levels. In neurological and neurodegenerative diseases, it was shown that activated microglia release glutamate suggesting their involvement in excitotoxicity, and hence neurodegeneration (Takeuchi et al. 2008). Inflammatory cytokines can cause neuronal death by stimulating excitotoxicity and/or apoptosis (Rossi et al. 2014) (Fig. 4).

It has been found that IL-1β may enhance synaptic glutamatergic neurotransmission (Rossi et al. 2012) and excitotoxicity leading to neuronal damage (Forder and Tymianski 2009). Although IL-1 has not shown neuronal toxicity in culture and can enhance neuronal survival by augmenting GABAergic inhibition (Simi et al. 2007), it can induce neuronal death by other signaling mechanisms. It can act directly on neurons by activating Src kinase which phosphorylates the NMDAR2A/B subunit, increasing calcium influx and neuronal damage (Viviani et al. 2003). Moreover, Takahashi et al. (2003) found that IL-1b destroyed oligodendrocytes co-cultured with astrocytes by a glutamate receptor-dependent mechanism. IL-1 may also cause neuronal death indirectly through caspase-dependent apoptosis induced by the IL-1RI astrocytic receptor in neuronal-glial cultures (Thornton et al. 2006). It may also increase neurotoxicity by enhancing leucocyte recruitment by its actions on the vascular endothelium (Konsman et al. 2007) and damage the neurovascular unit by generating ROS and matrix metalloproteinases (MMPs). Additionally, IL-1β induced the production of nitric oxide synthase (NOS) and arachidonic acid (AA) which participate in the pathology of neurological disorders (Sung et al. 2004). AA can cause glutamate accumulation in the synaptic cleft by enhancing its release (Vázquez et al. 1994), and reducing its uptake (Barbour et al. 1989).

In addition, there is a functional interaction between IL-6 and glutamate. It has been shown that IL-6 decreased glutamate excitotoxicity during hypoxic insults and in primary cortical neuronal cultures and inhibited glutamatergic excitatory transmission by upregulation of adenosine A1 receptor mRNA in hippocampal slices (Biber et al. 2008).

It has also been reported that IL-17 enhances short-term memory and synaptic plasticity by modulating the ratio of the glutamatergic synapses AMPA/NMDA (Ribeiro et al. 2019). Ribeiro et al. (2019) demonstrated that IL-17 A released from gd T cells in the healthy meninges, promoted short-term memory by supporting glutamatergic synaptic plasticity of CA1 hippocampal neurons. The authors reported the success of IL-17 neutralization to prevent short-term memory impairment and hippocampal glutamatergic dysfunction in early AD stages while prolonged anti-IL-17 infusion delayed cognitive impairment, and reduced peripheral inflammation in later AD stages. They proposed that elevated IL-17 levels early in AD disrupted synaptic functions and short-term memory in the 3xTg-AD mouse model. IL-17 can act directly on cortical glutamatergic neurons (Alves de Lima et al. 2020) and interneurons (Chen et al. 2017), and indirectly on glial cells, to amplify neuronal responses by promoting brain-derived neurotrophic factor (BDNF) production (Ribeiro et al. 2019).

Several studies have shown that IL-10 has a direct neuroprotective effect against glutamate-induced excitotoxicity by blocking the activity of proapoptotic proteins (Bachis et al. 2001) and inhibiting the formation of inflammatory cytokines as TNF, IL-1β and prostaglandins (Lee et al. 2002). IL-10 can alter long-term potentiation, confirming the participation of this interleukin in glutamatergic transmission (Kelly et al. 2001). The deficiency of IL-10 decreased mGlu-receptor 1a/b expression, and NMDA-receptor sensitivity to polyamines and altered nitric oxide glutamate-dependent production (Koriauli et al. 2014). This cytokine exerted neuroprotective effects by inhibiting inositol-3-phosphate receptor (IP3R)-sensitive release of Ca^2+^ from internal storage sites after repeated stimulation of NMDA receptor. In addition, it activated the canonical signaling pathways, (Turovskaya et al. 2012).

IL-10 provided trophic and survival effects against glutamate-dependent excitotoxicity (Zhou et al. 2009). IL-10 inhibited NADPH oxidase activity reducing neuroinflammation and increasing neuronal survival (Park et al. 2007).

IL-4 treatment of wild-type neurons shifted the balance toward increased frequency of inhibitory currents, suggesting that IL-4 can modulate the balance toward increased inhibition, thereby reducing the risk of hyperexcitation. Finally, IL-4Rα deficiency led to deficits on the synaptic and neuronal network level causing behavioral abnormalities with overall hyperactivity or restlessness.

## Role of interleukins in oxidative stress

Activation of microglia and microglial-induced oxidative stress play a critical role in AD pathogenesis and neurodegeneration (Block and Hong 2005). Reactive oxygen species (ROS) are produced normally during oxidative metabolism and mitochondrial respiration. However, they are also formed in pathological conditions accompanied by neuroinflammation. ROS induce neuronal damage leading to numerous neurological diseases (Jenner and Olanow 2006). Their over-production may be related to the high levels of IL-1 (Brabers and Nottet 2006). Sustained oxidative stress and neuroinflammation are cross-linked and may affect each other (Lugrin et al. 2014), and are considered main features of neurodegenerative diseases. Mitochondrial dysfunction causes neurons to release activating cytosolic factors to neighboring astrocytes and microglia which in turn secrete RNS, ROS and proinflammatory cytokines to enhance the inflammatory process and eventually lead to neurodegeneration and neuronal death (Fischer and Maier 2015).

Overexpression of IL-1β was reported to be caused mainly by oxidative stress (Brabers and Nottet 2006) and excitotoxicity (Reynolds and Hastings 1995). Superoxide dismutase (SOD)−1 reduction in PC12 cells resulted in increased ROS production and IL-1β expression (Troy et al. 1996). In addition, microglia, activated by Aβ, released IL-1β which potentiated Aβ deposition and enhanced the production of ROS and RNS by the activated microglia. This promoted oxidative stress, and IL-17 formation by induced Th17 cells, which attracted neutrophils to actively invade mice CNS with high levels of APP (Zenaro et al. 2015). IL-17 was suggested to increase neuronal autophagy and cause neurodegeneration (Wang et al. 2019).

In AD, activated microglia and astrocytes accumulate around extracellular Aβ-plaques and damaged or dead neurons (Ojala et al. 2009; Perry et al. 2010) releasing pro-inflammatory cytokines and ROS (Morales et al. 2014). ROS damage all cell membranes, causing modifications in proteins, lipids, and DNA and disrupting the functions of membrane receptors, and intracellular signaling pathways. Pro-inflammatory interleukins, as IL-18, together with oxidative stress can enhance Aβ generation (Opazo et al. 2002) which in turn promotes ROS by penetrating mitochondria; the toxicity of Aβ could be blocked partly by antioxidants (Wang et al. 2016). Oxidative stress can stimulate the expression of kinases, which result in tau hyperphosphorylation and the formation of NFTs (Ojala et al. 2008).

IL-18 is a potent activator of oxidative stress (Sutinen et al. 2014) and IL-18 binding protein can alleviate oxidative stress (Gonul et al. 2016). Another important feature of inflammation is the “Warburg effect” in which the activation process affects aerobic glycolysis as reported in cancer (Capello et al. 2016). The ‘inverse Warburg effect’ in which ROS induce oxidative phosphorylation is linked to AD. IL-18 affects glucose metabolism by enhancing glycolytic γ-enolase (ENOG) and reducing α-enolase (ENOA) (Sutinen et al. 2014). ENOs form homo- or heterodimers, and are accompanied with AD (Butterfield and Lange 2009).

IL-4 and IL-13 suppressed the formation of ROS (Cash et al. 1994) and pro-inflammatory molecules, as IL-1β, IL-6, TNF-α, and iNOS (Szczepanik et al. 2001). IL-10 abolished the stimulation of superoxide dismutase activity and ROS generation by IL-1β. In addition, the inhibition of LTP induced by H_2_O_2_ was blocked by IL-10, indicating the ability of IL-10 to cause shedding of type I receptor of IL-1 (Kelly et al. 2001).

Glutamate toxicity may increase the influx of Ca^2+^ into neurons and the subsequent generation of oxidative/nitrosative species that damage neurons (Nicholls 2008; Dorsett et al. 2017). This increases NOS transcription (Zhang et al. 2015) leading to increased NO and peroxynitrite production which damage lipids, proteins, DNA and different cellular structures causing neuronal death (Burda et al. 2016).

It has been reported that the chronic administration of NMDA results in up-regulation of proinflammatory mediators as TNF-α, IL-1β and glial fibrillary acidic protein (GFAP) and iNOS expression in rat brain (Keleshian et al. 2014). This suggests that there is a link between neuroinflammation and excitotoxicity involving the release of NO and upregulation of iNOS (Persichini et al. 2016). After injury, microglial cells migrate to the site of injury in response to the energy released by damaged neurons (Paquet et al. 2017). They stimulate iNOS which produces NO that plays a dual role in the pathogenesis of neurological diseases (Deng-Bryant et al. 2008).

## Therapeutic targets

The neurodegenerative diseases of the CNS present a great therapeutic challenge. Understanding the role of interleukins in neuroinflammatory mechanisms contributing to neurodegenerative diseases may assist in developing potent therapeutic agents (Maguire-Zeiss and Federoff 2009). Among the inflammatory cytokines, IL-1β has gained special attention due to its role in the pathogenesis of AD. The IL-1 system was modified using several targets (Bresnihan 2001). Blocking IL-1β was able to prevent cognitive impairment and reduce tau pathology and Aβ synthesis in a mouse model of AD (Kitazawa et al. 2011). The authors found that blocking IL-1 signaling could alter pro-inflammatory responses by decreasing NF-kB activity, tau pathology and some oligomeric and fibrillar forms of Aβ in 3xTg-AD mice.

The use of proinflammatory cytokine modulators as melatonin, which is normally secreted by the mammalian pineal gland, showed anti-inflammatory activity. Melatonin inhibited the local neuroinflammation induced by glial activation resulting from IL-1β thereby improving cognitive disruption in rats (Shen et al. 2007). Moreover, it attenuated the inflammatory response, and reduced the Aβ-induced production of the proinflammatory cytokines TNF-α, IL-1β, and IL-6 by 50% (Rosales-Corral et al. 2003). Although melatonin has shown success as an adjuvant in AD treatment (Spuch et al. 2010; Yang et al. 2011), negative results were obtained after melatonin administration in AD patients (Singer et al. 2003).

Green tea flavonoids like epigallocatechin gallate may enhance downregulation of innate immunity by direct free radical scavenging (Hashimoto et al. 2003) and reduction of proinflammatory cytokine production including IL-1β, prostaglandin E2, and TNF-α (Zheng et al. 2008). These studies suggested that modulators of pro-inflammatory cytokines may be developed as potential therapy for AD. Ellagic acid (EA) is another neuroprotective antioxidant drug (Ahmed et al. 2016). It reduces IL-17 release by brain CD3 + T through two mechanisms; maintaining BBB integrity and suppressing microglial migration which are the major sources of brain cytokines (Sanadgol et al. 2017).

Several studies reported that inhibiting the over-production of proinflammatory interleukins improved AD in animal models. Rojanathammanee et al. (2013) found that T-cell and microglial activation were inhibited after treatment of transgenic AD mouse model with pomegranate polyphenols extract. Moreover, Zhang et al. (2013b) found that atorvastatin improved learning and memory and reduced neuronal loss by inhibiting the production of hippocampal IL-1β, IL-6, and TNF-α in an AD rat model induced by Aβ1–42. In addition, matrine improved cognitive deficits and restored the balance of Th17/Treg cytokines in the same AD rat model.

On the other hand, the attenuation of neurodegeneration by IL-10 was supported by AD studies in transgenic mouse models, in which IL-10 could reduce neuroinflammation, promoting neurogenesis and improving spatial cognitive deficits (Guillot-Sestier et al. 2015). Accumulating evidence suggested that post-menopausal administration of estrogens increased IL-10 release by microglia which contributed to AD prevention (Henderson 2014; Maki and Henderson 2016). In addition, treatment with the natural polyphenol resveratrol induced anti-inflammatory effects by upregulating IL-10 levels and IL-10 gene expression, leading to neuroprotection (Cianciulli et al. 2015). Thus, Bagyinszky et al. (2017) suggested that IL-10 could be used as a potential treatment for AD, since it induced downregulation of pro-inflammatory cytokine expression and amyloid reduction.

Another drug, montelukast, which is a cysteinyl leukotriene receptor 1 antagonist, inhibited TNF-α and IL-1β and thus alleviated memory impairment caused by Aβ. The neuropathological role of IL-6 has been established in AD patients and animal models. Minocycline, a tetracycline semisynthetic derivative, showed anti-inflammatory activity, reducing the astrocyte-released pro-inflammatory cytokines IL-6 and TNF-α (Familian et al. 2007; Garwood et al. 2010). Fu et al. (2016) demonstrated that peripheral administration of IL-33 alleviated AD pathology by increasing microglial degradation and phagocytosis of Aβ in AD mouse models. The authors suggested that that IL-33 can be used as a novel therapeutic strategy for AD (Table 1).

**Table 1:** Interleukins with pro- and/or anti-inflammatory roles in Alzheimer’s disease

	Pro-inflammatory	Anti-inflammatory	Mechanism of Action
IL-1β	Kitazawa et al. 2011	Ghosh et al. 2013	-enhances amyloid precursor protein (APP) expression -exaggerates tau phosphorylation and neurofibrillary tangle formation -impairs the ability of microglia to clear Aβ -regulates the degradation of microglial-dependent plaque -enhances the cleavage of non-amyloidogenic APP
IL-2	Dansokho et al. 2016	Alves et al. 2017	-enhances cortical plaque-associated microglial activation -increases Treg activation and improved AD pathology
IL-3	Kiddle et al. 2012	McAlpine et al. 2021	-correlated with risk and severity of AD -induces rapid microglial mobilization -microglial accumulation around Aβ
IL-4	Nam et al. 2012	Kiyota et al. 2010	-potentiates Aβ-induced inflammation by generating ROS and proinflammatory cytokines -reduces Aβ, glial activation, and neurogenesis -restores cognitive functions
IL-6	Spooren et al. 2011	Chakrabarty et al. 2010	-participates in early-stage amyloid plaque formation -underlies synaptic loss and learning deficits -contributes to the processing and production of APP and NFT formation by Tau hyperphosphorylation suppresses Aβ deposition in APP transgenic mice
Il-8	Alsadany et al. 2013		-associated with a reduced performance in cell cognition tasks
IL-9	Upadhya 2020		-up-regulated in AD
IL-10	Mun et al. 2016	Kiyota et al. 2012	-reduces synaptic and cognitive impairments -enhances Aβ clearance in knockout mice enhances neurogenesis and cognition
IL-12	Schneeberger et al. 2021		-causes IL-12/IL-23 neuroinflammation, which preferentially targets oligodendrocytes and neurons
IL-13	Nam et al. 2012	Kawahara et al. 2012	-potentiates Aβ-induced inflammation by generating ROS and proinflammatory cytokines -decreases amyloid deposition -promotes spatial memory and learning in transgenic mice
IL-15	Bishnoi et al. 2015		-correlated with dementia in AD
IL-17	Cao et al. (2023)	Yang et al. 2017	-promotes the formation of Aβ plaques -enhances the secretion of TNF-α by microglia -attenuated the phagocytic activity of microglia -reduces amyloid angiopathy -relieves memory and learning impairments
IL-18	Sutinen et al. 2012		-increases Aβ production -increases tau hyperphosphorylation -modulates anti-apoptotic B-cell lymphoma-extra-large (Bcl-xL) protein levels
IL-23	Nitsch et al. 2021		-neuroinflammation and formation of plaques in AD models
IL-33	Xiong et al. (2014)	Fu et al. 2016	-aggravates neuroinflammation -decreases soluble Aβ levels and the deposition of amyloid plaque -reverses the deterioration in synaptic plasticity and cognitive functions -restores the phagolysosomal activity of microglia -enhances Aβ clearance

### Current approaches to target interleukins in AD

Specifically, the use of antibodies which inhibit proinflammatory interleukins has succeeded in developing potential therapies for numerous diseases (Nitsch et al. 2021). Recently, anti-cytokine drugs that target interleukins in AD have been developed. Anakinra serves as an IL-1β receptor antagonist that decreases neuroinflammation, cognitive impairment, and tau phosphorylation, while canakinumab neutralizes IL-1β and prevents its proinflammatory effects (Melchiorri et al. 2023). Tocilizumab, a monoclonal antibody against IL-6 or IL-6R signaling, improved learning and spatial memory and reduced Aβ (Elcioglu et al. 2016). However, it inhibited both the classical and trans-signaling pathways (Nishimoto and Kishimoto 2008), thus affecting both inflammation and anti-inflammation. To overcome this drawback, an alternative strategy was developed based on the selectivity of the gp130 soluble form to block trans-signaling (Jostock et al. 2001), thus decreasing the side effects of treatment (Choy et al. 2020). Ustekinumab inhibits the p40 subunit of IL-12 and IL-23 (Cingoz 2009) and can thus represent an effective AD therapy. However, despite its promising efficacy in mice, the drug has not yet been practiced in clinical trials.

The uptake of blocking anti-IL-17 antibody was reported to antagonize the Ab1 − 42 injected into the cerebral ventricles of CD1 mice and ameliorate cognitive impairment and neuroinflammation (Cristiano et al. 2019). Additionally, anti-IL-17 A antibody may interfere with neutrophil recruitment to the brain of AD patients by inhibiting IL-8 production by neutrophils (Keijsers et al. 2014). It has been suggested that administration of anti-IL-17 A and anti-IL-23 antibodies to patients with AD may prevent neutrophil infiltration into the brain and inhibit AD progression (Katayama 2020).

## Conclusion

Interleukins play critical roles in both neurodegeneration and neuroprotection. Most of the interleukins exhibit dual effects as pro-inflammatory or anti-inflammatory depending not only on the stage of AD but also on the interactions between different interleukins and the cell types activated. This causes the manipulation of interleukins and their receptors to be challenging since our knowledge of the complex role of different interleukins in AD is not complete. The use of the beneficial anti-inflammatory interleukins and the control of the deleterious pro-inflammatory interleukins as therapeutic strategies in AD may be over simplistic. Instead novel therapeutic approaches must have the ability to balance the pro-inflammatory and anti-inflammatory interleukin signaling and direct microglial activity towards the protective phenotype. Thus, extensive research is required to clarify the mechanisms underlying the actions of interleukins and their relations to neurochemical and neurobehavioral functions to produce a complete picture of the neuro-immunological architecture of the CNS in healthy and AD patients. Future studies should emphasize not only on how they contribute to AD pathology and neuroinflammation but should also take into consideration their relation to excitotoxicity, oxidative stress and other apoptotic mechanisms. The aspects of interleukins and their contribution to AD will remain an interesting research field that merits extensive investigation.

## Data Availability

No datasets were generated or analysed during the current study.
